# *PyunBBX18* Is Involved in the Regulation of Anthocyanins Biosynthesis under UV-B Stress

**DOI:** 10.3390/genes13101811

**Published:** 2022-10-07

**Authors:** Qin Zhang, Dongxiao Ma, Zhixu Hu, Dan Zong, Chengzhong He

**Affiliations:** 1 College of Life Sciences, Southwest Forestry University, Kunming 650224, China; 2 Key Laboratory for Forest Genetic and Tree Improvement and Propagation in Universities of Yunnan Province, Southwest Forestry University, Kunming 650224, China; 3 Key Laboratory of Biodiversity Conservation in Southwest China, State Forestry Administration, Southwest Forestry University, Kunming 650224, China

**Keywords:** *BBX*, *Populus yunnanensis*, UV stress, anthocyanins, gene family, B-box

## Abstract

(1) Background: *Populus yunnanensis* Dode (*P. yunnanensis*) grows in the low-latitude and high-altitude areas of southwest China. In low-latitude and high-altitude areas, plants suffer from the high intensity of UV-B (ultraviolet-b) radiation, and they have a complete regulation system to adapt to the environment of the high UV-B radiation. As natural antioxidants, anthocyanins play an important role in scavenging free radicals. *BBX* (B-box) genes are involved in anthocyanins biosynthesis. (2) Methods: By exploring the gene structure and motifs of *PyunBBX* genes (genes of *P. yunnanensis BBX* family) and the evolutionary relationship between *PyunBBX* genes and other species *BBX* genes, six *PyunBBX* genes that responded to UV-B and participated in anthocyanins biosynthesis were screened. *BBX,* with the potential to regulate anthocyanins biosynthesis, was further investigated by anthocyanins content determination and RT-qPCR (real-time quantitative polymerase chain reaction); (3) Results: After 7 days of UV-B treatment, anthocyanins were significantly accumulated, and the expression of *PyunBBX18* was up-regulated for 7 days. The expression of *PyunBBX12* was inhibited by UV-B treatment. By analyzing the RNA-seq data of leaves and bark of *P. yunnanensis*, we found that *PyunBBX18* was highly expressed in leaves and young bark; (4) Conclusions: These results showed that *PyunBBX18* and *PyunBBX12* may be involved in the response process of UV-B stress, in which *PyunBBX18* may regulate the anthocyanins biosynthesis to resist UV damage.

## 1. Introduction

Transcription factors (TFs) play an important role in plant growth and development and stress defense. *BBX* is a kind of TF containing a zinc finger structure that mediates and regulates many biological processes in plants, including photomorphogenesis, flowering regulation, and response to biological and abiotic stresses [1]. The N-terminal of the BBX protein contains one B-box-conserved domain or two B-box-conserved domains, namely B-box1 and B-box2. Some *BBX* have a CCT (CONSTANS, CO-like, and TOC1) domain at the C-terminal. The B-box conserved domain is involved in protein interaction and transcriptional regulation, while the CCT domain is mainly involved in nuclear transport and transcriptional regulation [2,3].

Over the years, research has shown that the majority of *BBX* gene family members can interact with *HY5* (ELONGATED HYPOCOTYL5, an important TF of light signal) or regulate transcription of *HY5*, thereby regulating photomorphogenesis and anthocyanins biosynthesis in plants [4,5,6,7,8,9,10]. For instance, *AtBBX21* binds to T/G-box in the *AtHY5* promoter through its second B-box domain, thereby regulating the expression of *AtHY5* and HY5-regulated genes to promote photomorphogenesis in plants [5]. Under UV-B treatment, the module *SIBBX20/21-SIHY5* could activate the transcription of *SIHY5*, and the excessive accumulation of SIHY5 protein inhibited its transcription, forming a negative feedback loop to maintain the level of *SIHY5* in plants, thus regulating the photomorphogenesis of tomato [11]. *PtrBBX23* showed a high expression response in *Populus trichocarpa* (*P. trichocarpa*) after different treatments, including high light, blue light, and UV-B radiation. The interaction experiment proved that the transcription of *MYB115/119* and structural genes (*CHS, F3H*), which are associated with anthocyanins biosynthesis, was activated by *PtrBBX23* bound to their promoters directly, and the interaction between *PtrBBX23* and *HY5* enhanced the activation activity of *PtrBBX23*, thus promoting the accumulation of proanthocyanidins and anthocyanins [12]. *MdBBX22* can enhance the binding of *HY5* with *MdMYB10* and *MdCHS* by interacting with *HY5*, thereby promoting anthocyanins biosynthesis of apple fruit response to UV-B signal [13], and a similar mechanism exists in *PpBBX16* [10] and *MdBBX20* [14].

Anthocyanins are plant pigment with natural activity, and it is a kind of secondary metabolite produced in plants. It is distributed in flowers, leaves, fruits, and other organs. Biosynthesis and accumulation of anthocyanins play an important role in plant adaptation and resistance to harsh environment and can enhance plant resistance to biological and abiotic stresses [15,16,17,18,19,20,21,22,23,24]. Anthocyanin accumulation is not only affected by plants’ own factors but also by external environmental factors, among which light signal is one of the most important environmental factors affecting anthocyanins biosynthesis [25].

*P. yunnanensis* is an endemic *Tacamahaca* in China and a representative species of *Populus* in low-latitude and high-altitude areas of southwest China. It is mainly distributed in high-altitude mountains from 1600 to 3200 m, which have suitable characteristics of strong adaptability, rapid growth, and easy rooting, and it plays an important role in forestry production, afforestation, and environmental protection [26]. Previous studies have shown that the UV-B radiation intensity will increase by 10–16% in every 1000 m rise in altitude [27]. So, in high-altitude areas, *P. yunnanensis* suffers from the high intensity of UV-B radiation, and it has a complete regulation system to adapt to the environment of the UV-B radiation. As natural antioxidants, anthocyanins play an important role in scavenging free radicals. It can protect plant tissues from excessive UV-B radiation, and enhanced UV-B can promote and induce the biosynthesis of anthocyanins [28,29,30]. However, the fundamental research on molecules of *P. yunnanensis* in response to UV-B stress is limited. Therefore, further molecular studies are needed to explain the underlying mechanism of adaptation to UV-B radiation in *P. yunnanensis*. In this study, we identified 43 *PyunBBX* genes and investigated their structural domains and the effects of UV-B irradiation on the *BBX* gene expression and anthocyanins content in *P. yunnanensis*. This work will be helpful for future studies of *BBX* gene functions under UV-B radiation stress in *P. yunnanensis.*

## 2. Materials and Methods

### 2.1. Identification of BBX Gene Family Members in P. yunnanensis

The genome at the chromosome level of *P. yunnanensis* was obtained from our laboratory group (Southwest Forestry University, Kunming 6500224, China). In order to identify the *BBX* in *P. yunnanensis* genome, firstly, the 32 members of BBX proteins sequences in *Arabidopsis thaliana* (*A. thaliana*) genome were downloaded from the TAIR 10 (https://www.arabidopsis.org/, accessed on 1 December 2021) database [3]. The 32 proteins sequences were used to search the BBX family members in *P. yunnanensis* by Blastp (E-value < 1E-05) [31]. The profile of the hidden Markov model (HMM) for the B-box domain (Pfam00643) and CCT domain (Pfam06203) was obtained from Pfam (https://pfam.xfam.org/, accessed on 2 December 2021) [32]. The hmmsearch program of the HMMER 3.0 software was used to search for the putative *BBX* gene family members in the *P. yunnanensis* genome, and the E-value was set to 1E-05 [33]. If a gene had multiple transcripts, the longest transcript was selected as the representative protein sequence for further analysis. After removing the duplicate for candidate *BBX* gene family members, the reserved protein sequences of *BBX* gene family members were submitted to the SMART database (http://smart.embl-heidelberg.de/, accessed on 3 December 2021) [34], CDD (Conserved Domain Database, https://www.ncbi.nlm.nih.gov/Structure/cdd/cdd.shtml, accessed on 3 December 2021) [35], and InterPro database (http://www.ebi.ac.uk/interpro/, accessed on 3 December 2021) [36] in order to analyze whether it contained unique B-box conservative domain structure. The B-box conservative domain structure of candidate genes was identified as the *BBX* gene family members of *P. yunnanensis*.

### 2.2. Analysis of Basic Physicochemical and Subcellular Localization of PyunBBX Genes

The molecular weight (kDa) and isoelectric point (PI) of each BBX protein were predicted by ExPASy (http://www.expasy.org/tools/, accessed on 4 December 2021) [37]. The online software Plant-mPLoc (http://www.csbio.sjtu.edu.cn/bioinf/plant-multi/, accessed on 4 December 2021) was used to predict the subcellular localization of *PyunBBX genes* [38].

### 2.3. Gene Structure and Motif Analysis of PyunBBX Genes

The length and location of exons and introns of *PyunBBX* genes were obtained from the *P. yunnanensis* genome for further analysis. Each sequence of *PyunBBX* genes was submitted to MEME (https://meme-suite.org/meme/tools/meme, accessed on 18 December 2021) [39]. In the conservative base sequence analysis, the maximum was set to 10, the mode was selected as zoops, and the default values of other parameters were used. Finally, the results were visualized by TBtools [40] and WebLogo (http://weblogo.berkeley.edu/logo.cgi, accessed on 20 December 2021).

### 2.4. Cis-Acting Elements Analysis

The promoter-upstream region (~2000 bp) sequences were extracted from the genomic DNA sequence of *P. yunnanensis,* and the sequences were submitted to the PlantCARE website (http://bioinformatics.psb.ugent.be/webtools/plantcare/html/, accessed on 2 January 2022) [41] for cis-acting element prediction. Excel was used to sort out and simplify the analysis results, and the cis-acting elements with the same biological functions were labeled with the same label notes.

### 2.5. Chromosomal Location, Gene Duplication, and Synteny Analysis

The chromosome location image of the *PyunBBX* genes was drawn using the TBtools software according to the physical positions of *PyunBBX* genes on the *P. yunnanensis* chromosomes. All the BBX amino acid sequences of *A. thaliana, P. trichocarpa,* and *P. yunnanensis* were included in a local database using DIAMOND [42]. The *P. trichocarpa* genome (version = 3.0) was derived from the Phytozome database (https://phytozome-next.jgi.doe.gov/info/Ptrichocarpa_v3_0, accessed on 15 March 2022). The BBX protein sequences of *P. yunnanensis* were used as queries to search the above-mentioned database with an E-value of 1E-05. The blast results were analyzed by the MCScanX (Multiple Collinear Scan Kit) [43] and TBtools to determine and analyze the duplication and synteny of *BBX* genes. The Ks (synonymous substitution rate) and Ka (nonsynonymous substitution rate) values of collinearity pairs in *PyunBBX gene*s were estimated by the KaKs_calculator [44] procedure in TBtools. The divergence time was calculated with the formula T = Ks/2r, with Ks being the synonymous substitutions per site and r being the rate of divergence for nuclear genes from plants. The r was taken to be 1.5 × 10^−8^ synonymous substitutions per site per year for dicotyledonous plants [45].

### 2.6. Evolutionary Analysis of BBX Gene Family Members

The molecular evolutionary genetic analysis tool MEGA (Molecular Evolutionary Genetics Analysis) version 7.0.20 [46] was used for multiple sequence alignment and evolutionary analysis. The ClustalW program of MEGA was firstly used for multi-sequence alignment of the BBX amino acid sequences. Then, the gaps were manually sheared, and conservative region sequences were reserved. The maximum likelihood (ML) method was selected to construct the phylogenetic tree for *PyunBBX* genes, and the neighbor-joining (NJ) method constructed a phylogenetic tree for *BBX* genes of multispecies. Bootstrap was set to 1000.

### 2.7. Plant Materials, Growth Conditions, and UV Treatment

The plant material was from the branches of *P. yunnanensis* that had grown for one year in the greenhouse of Southwest Forestry University. They were pruned into cuttings of about 15 cm, and cuttings were made in March 2022. The cuttings were induced when they had reached 5–6 leaves. The cuttings of the same growth were placed in the constant temperature incubator (temperature: 25℃, humidity: 70%, light intensity: 90 µmol·m^−2^·s^−1^, photoperiod: 12 h light/12 h dark) for cultivation, a UV lamp (Telipu, Beijing Zhongyi Boteng Technology Company, power: 8 W, radiation intensity: 68 µW/cm^2^, wavelength: 280–320 nm) was placed 35 ± 1 cm above the cuttings, and UV irradiation was carried out for 12 h every day under normal light treatment. The cottage seedlings were sampled after UV treatment for 12 h, and the sampling time was 0, 1, 2, 3, 5, and 7 days. The control group was cottage seedlings that grew under normal light. The sampling sites were the second and third fully extended leaves. The samples were frozen with liquid nitrogen and immediately stored in the −80 °C refrigerator.

### 2.8. Total RNA Isolation and cDNA Synthesis

Total RNA was isolated from the leaf of *P. yunnanensis* using the E.Z.N.A.^@^ Plant RNA kit (Omega Bio-tek Inc., Norcross, USA) according to its manual. The quality of RNA was evaluated with K5800C (KAIAO, Beijing, China). A total of 500 ng RNA of each sample was used for 1st strand cDNA synthesis using Hifair^®^ Ⅲ Reverse Transcriptase (YEASEN, Shanghai, China) according to the manufacturer’s protocols. cDNA was diluted 8-fold for RT-qPCR analysis.

### 2.9. Quantitative Real-Time PCR

Primers were designed based on CDS sequences for real-time PCR by using NCBI primer-blast (Table S1). The internal reference gene was HIS (histone) [47]. Real-time PCR application was carried out in a LightCycler^®^ 96 Real-Time PCR Detection System (Roche, Hercules, Switzerland) with Hieff UNICON^®^ Universal Blue qPCR SYBR Green Master Mix (YEASEN, Shanghai, China). A total of 20 μL reaction system contained 10 μL Blue qPCR SYBR Green Master Mix, 1 μL cDNA samples, 0.4 μL of each primer (1 μM), and 8.2 μL ddH2O. The PCR thermal cycle conditions were as follows: denaturation at 95 °C for 2 min, 45 cycles of 95 °C for 10 s, and 56 °C for 30 s. Fluorescence intensities were measured for RT-qPCR at the end of each cycle. A melting curve (1 cycle of 95 °C for 10 s, 65 °C for 10 s, and 97 °C for 1 s) was performed directly to check for specific amplification. The relative gene expression was calculated by using the 2^−^^△△^^Ct^ method [48], and the experiments were performed in triplicate technological repeats.

### 2.10. Extraction and Determination of Anthocyanins

Determination of anthocyanins content used ethanol/hydrochloric acid assay [49]. Fresh leaves were cut into pieces and weighed with 0.1 g, placed in a 10 mL centrifuge tube, and 10 mL hydrochloric acid ethanol (0.1 mol/L) solution was added to the tube. It was warmed at 60 °C for 30 min in a water bath. Then, the supernatant was transferred to a 25 mL volumetric flask after cooling. Then, 5 mL hydrochloric acid ethanol solution was added to the tube and continued water bath for 15 min. This step was repeated three times, and the supernatant was combined. Finally, the volume was fixed to 25 mL, and the absorbance value (Abs) was measured at 530 nm. Substitute the measured results into the following formula for calculation:

$$C=958^{-1}\times A\times V\times m^{-1}\times 10^{3}$$

C: anthocyanins content (mg/100 g), *A*: Abs (nm), *V*: metered volume (mL), *m*: mass (g), 958: empirical coefficient of Abs conversion into mass volume ratio, 10^3^: unit conversion coefficient.

### 2.11. Expression Patterns of BBX Family Members in P. yunnanensis by Rna-seq Data

Transcriptome data of leaves (PRJNA505895) and bark (PRJNA542544) at different mature sites (PRJNA506110) were downloaded from NCBI (National Center for Biotechnology Information). For raw data, fastQC (Version = 0.11.9) was used for quality inspection. Fastp (Version = 0.19.4) further filtered low-quality reads. The obtained high-quality clean reads were compared to the genome of *P. yunnanensis* using Hisat2 (version = 7.5.0). FeaturCounts (Version = 2.0.1) normalized FPKM (fragments per kilobase of exon model per million mapped fragments) values were obtained in the R language for expression pattern analysis. Heatmap (Version = 2.8.0) was used to draw the heat map of gene expression.

### 2.12. Data Analysis

Adobe Photoshop CS6 was used for image processing, and Adobe Illustrator 2020 was used for chart layout. The data obtained from the experiment were plotted and analyzed by Graphpad Prism 8.0.2 software.

## 3. Results

### 3.1. Analysis of The Basic Characteristics of PyunBBX Genes

Totally, 43 *PyunBBX* genes were identified from the *P. yunnanensis* genome. They were named *PyunBBX1* to *PyunBBX43* in the order of their position on chromosomes. They had different sequence lengths, resulting in different isoelectric points and molecular weights (Table 1). Amino acid sequences ranged in length from 184 aa (*PyunBBX10*) to 513 aa (*PyunBBX1*). The predicted molecular weight of the *BBX* gene was 12.11 kDa (*PyunBBX5*) to 59.46 kDa (*PyunBBX9*), and the theoretical isoelectric point was 4.12 (*PyunBBX30*) to 9.26 (*PyunBBX29*). Subcellular prediction showed that all of the *PyunBBX* genes were located in the nucleus.

**Figure 1:**
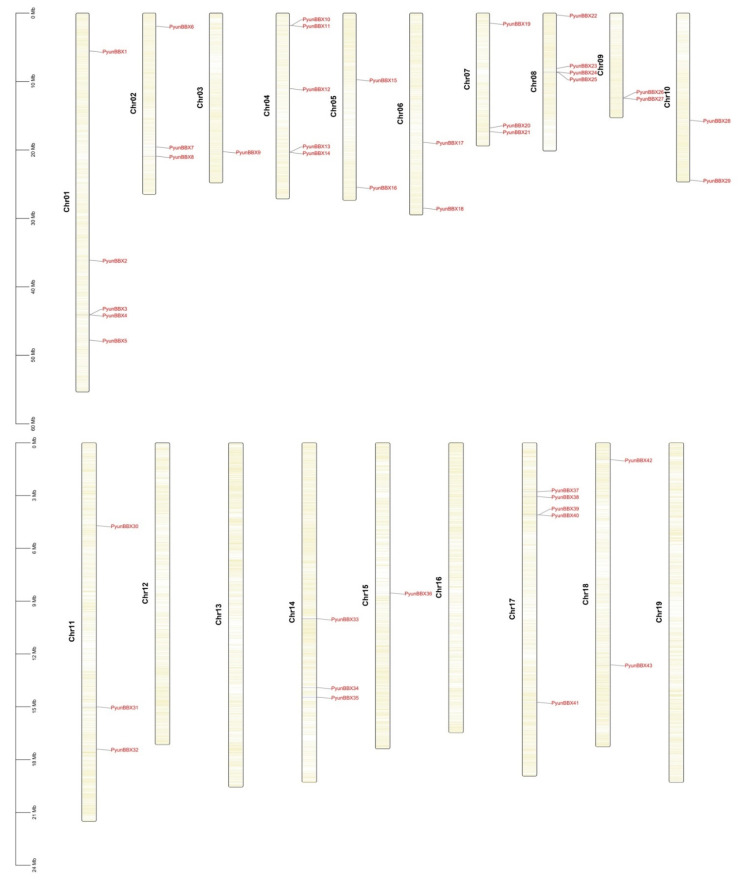
Chromosomes location of *PyunBBX* genes. The scale on the left is in megabases (Mb). The yellow lines within each chromosome represent gene density.

On 19 chromosomes of the *P. yunnanensis* genome, there were no *PyunBBX* genes on chromosomes 12, 13, and 19, and the other chromosomes (chromosomes 1–11 and chromosomes 14–18) had *PyunBBX* genes (Figure 1).

### 3.2. Gene Structure and Characteristics of Conserved Sequences of PyunBBX Gene Family Members

**Figure 2:**
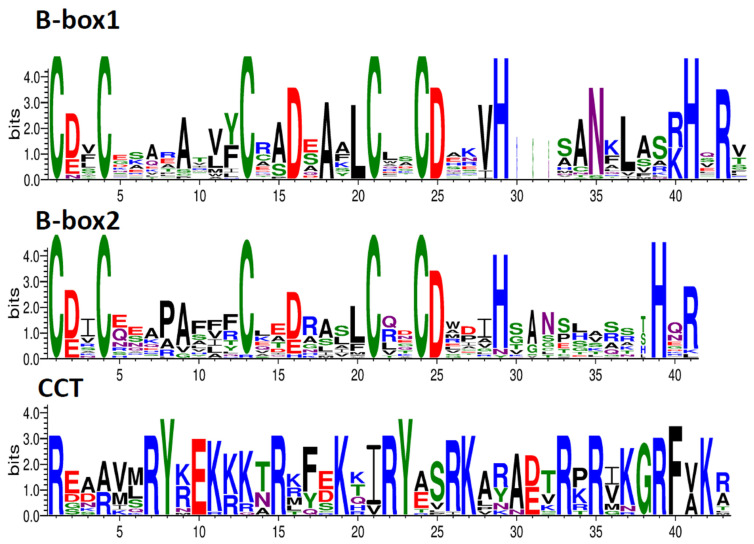
B-box1/B-box2 and CCT conserved domains and corresponding hidden Markov models (HMM) of BBX protein sequences in *P. yunnanensis*. The X-axis represents the conserved sequences of the domain. Conservation of each residue across all proteins is indicated by the height of each letter. The Y-axis is a scale of the relative entropy, which reflects the conservation rate of each amino acid.

**Figure 3:**
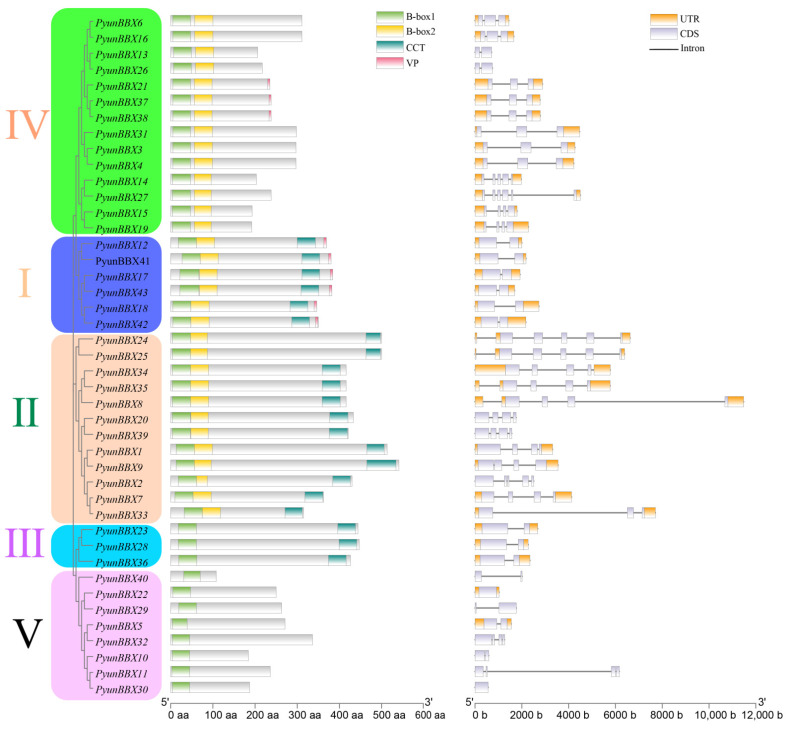
The phylogenetic tree and gene structures of *PyunBBX* *genes.* ML method with 1000 bootstrap replications to construct a phylogenetic tree. Starting from the left, the first scale below represents the number of amino acids (aa), and the second scale represents the number of bases (b).

The amino acid sequences of 43 members of the *P. yunnanensis BBX* gene family were analyzed, and the 3 most significant conserved sequences were extracted, namely B-box1, B-box2, and CCT (Figure 2, Figure 3, and Figure S1). B-box1 and B-box2 have a high similarity, with the structure of C_2_-X_2_-C-X_7, 8_-C-X_7_-C-X_2_-C-D-X_n_-H. Besides *PyunBBX5*, each of them has five conserved cysteine (Cys, C) residues adjacent to aspartic acid (Asp, D). Moreover, the Cys on both sides are (Leu, L) linked. Such a structure can combine with Zn^2+^ and form a “finger” structure through self-folding. It can be further combined with RNA, DNA, and protein to carry out gene regulation at the level of transcription and translation. Some *BBX* members also have a CCT domain, which is R-X_5_-R-Y-X-E-X_4_-R-X_3_-K-X_2_-R-Y-X_3_-K-X_5_-R-X-R-X_2_-G, containing a large number of arginine (Arg, R), followed by conservative tyrosine (Tyr, Y) and lysine (Lys, K), participating in the nuclear localization of the protein [50].

The structure of *PyunBBX* genes is shown in Figure 3. The results showed that most *PyunBBX* genes contained at least one intron and up to five introns. *PyunBBX* genes could be divided into five groups. It was consistent with the classification of other species. The first group contains 12 *PyunBBX* genes with 2 B-box domains and 1 CCT domain. The second group contains six *PyunBBX* genes with two B-box domains and one CCT domain. The third group contains three *PyunBBX* genes with a B-box domain and a CCT structure. The fourth group contains 14 members of *PyunBBX* genes with 2 B-box domains. The fifth group contains eight *PyunBBX* genes with a B-box domain. In addition, some *PyunBBX* genes (*PyunBBX12/17/18/21/37/38/41/42/43*) also have a VP motif, and the VP peptide motif in the gene can bind to the WD40 domain of *COP1* (CONSTITUTIVE PHOTOMORPHOGENIC 1) and interact with it to participate in the light response [51].

### 3.3. Collinearity Analysis

**Figure 4:**
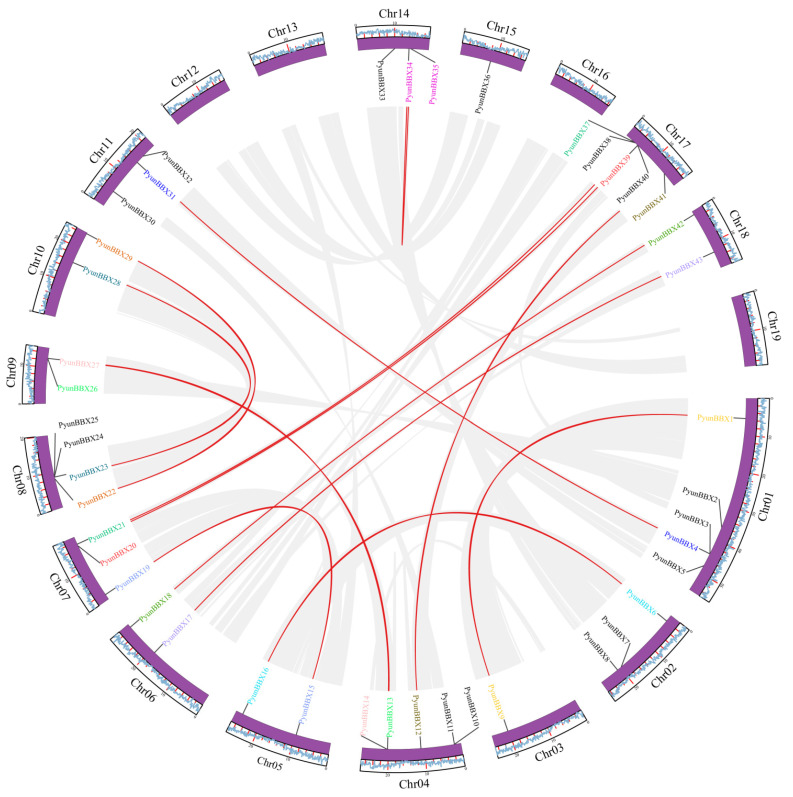
Gene duplication and synteny analysis of *PyunBBX* genes. The purple circles represent chromosomes. The outermost circle represents gene density, and the scale represents the number of bases (Mb). The 14 putative segmental-duplicated pairs of *PyunBBX* genes are linked by red lines, and the gene names are shown in the same color. The gray blocks represent replication events that occur on the chromosome of *P. yunnanensisg*.

The high sequence similarity between repetitive gene pairs indicated that they were likely involved in regulating similar biological processes. MCScanX was used to analyze the collinear blocks and gene duplication type. There were 14 gene pairs of *PyunBBX* genes, which were *PyunBBX1/9, PyunBBX6/16, PyunBBX15/19, PyunBBX13/26, PyunBBX14/27, PyunBBX18/42, PyunBBX23/28, PyunBBX22/29, PyunBBX4/31, PyunBBX34/35, PyunBBX21/37, PyunBBX20/39, PyunBBX12/41,* and *PyunBBX17/43*, and they belong to segmental in duplicated type (Figure 4, Table 2). The Ka/Ks values of 14 pairs of collinear blocks were all less than 1, indicating that these members may have been affected by purification selection in evolution. By calculating the divergence time of collinear blocks, the result showed that the average differentiation time of *PyunBBX* genes was 2.3 Mya (millions of years ago). Moreover, *PyunBBX22* and *PyunBBX29* differentiated at 3.92 Mya that they had the longest time of differentiation, and *PyunBBX34* and *PyunBBX35* differentiated at 0.24 Mya that they diverged more recently (Table 2).

**Figure 5:**
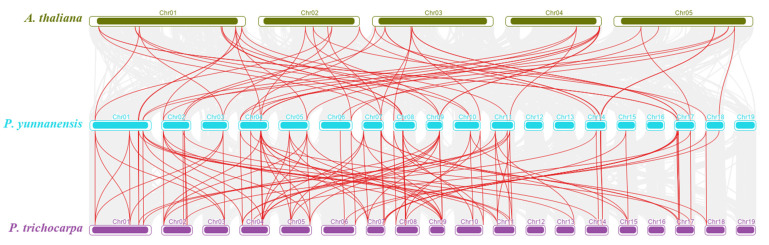
Synteny analysis between *P. yunnanensis* and the other two species. The putative collinear genes between *P. yunnanensis* and the other two species are marked in gray, while the syntenic *BBX* gene pairs are marked in red.

We constructed comparative syntenic maps between *P. yunnanensis* and the other two species (*P. trichocarpa* and *A. thaliana*) to analyze the similarities of *the BBX* gene among species. Syntenic maps revealed that 86 pairs of homologous genes were found between *P. trichocarpa* and *P. yunnanensis*, and 46 pairs of homologous genes were found between *P. yunnanensis* and *A. thaliana* (Figure 5).

### 3.4. Cis-Acting Elements of PyunBBX Genes Analysis

**Figure 6:**
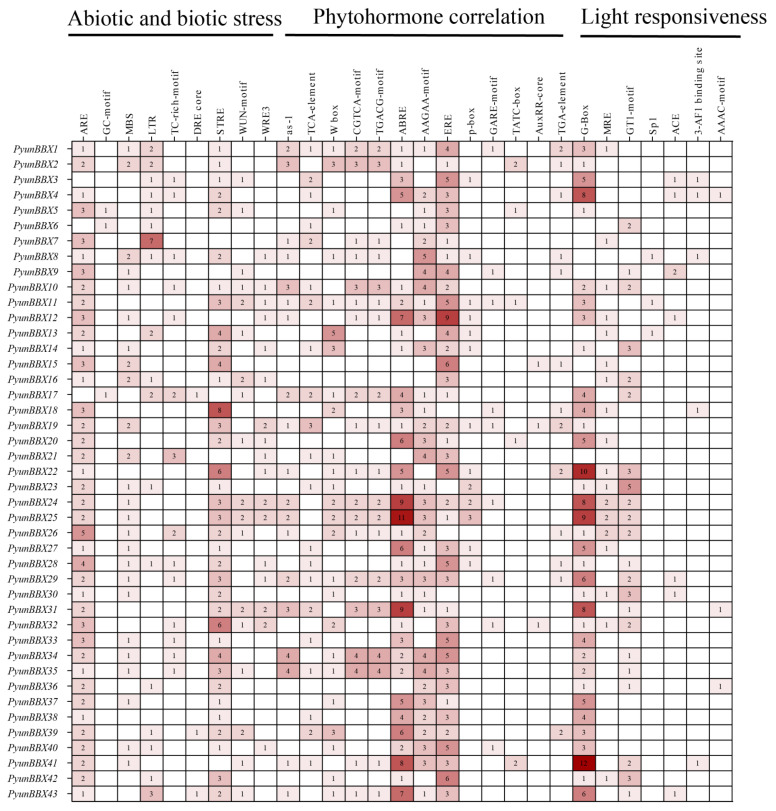
Analysis of cis-acting elements of *PyunBBX* genes promoters in *P. yunnanensis.* The number in the box represents the number of cis-acting elements. The shade of the color block depends on the number of cis-acting elements, and the deeper color, the more.

The promoter-upstream region (~2000 bp) sequences were extracted from the genomic DNA sequence of *PyunBBX* genes. The cis-elements of the *PyunBBX* gene family promoters were analyzed by using the PlantCARE database (Figure 6, Table S2). Each promoter of *PyunBBX* genes contains abiotic and biotic stress response elements (such as ARE, GC-motif, MBS, LTR, TC-motif, DRE-core, STRE, WUN-motif, and WRE3), phytohormone correlation (such as as-1, TCA-element, W box, CGTCA-motif, TGACG-motif, ABRE, AAGAA-motif, ERE, p-box, GARE-motif, TATC-box, AuxRR-core, and TGA-element), and light responsiveness (such as G-Box, MRE, GT1-motif, Sp1, ACE, 3-AF1 binding site, and AAAC-motif). Some *PyunBBX* genes had the cis-acting elements of the MYB binding site involved in light responsiveness, flavonoid biosynthetic genes regulation, and drought-inducibility (Table S2). This showed the functional diversity of the *PyunBBX* genes.

### 3.5. Expression Patterns of PyunBBX Genes in Leaf and Bark of P. yunnanensis by Rna-seq Data

**Figure 7:**
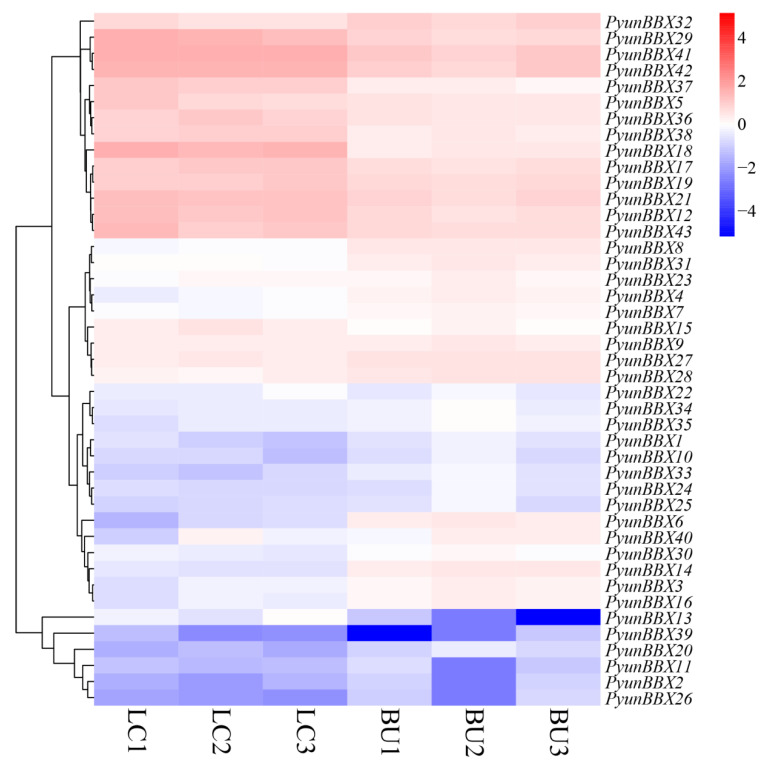
Expression patterns of 43 *PyunBBX* genes in leaf and bark. The FPKMs of 43 *PyunBBX* genes are taken as the logarithm with base 10 for standardization; the color patches of different colors indicate the expression levels of *PyunBBX* genes in the leaf and bark of *P. yunnanensis.* LC1/2/3: three biological repeats of leaves, BU1/2/3: three biological repeats of bark. The connecting lines on the left represent cluster analysis.

**Figure 8:**
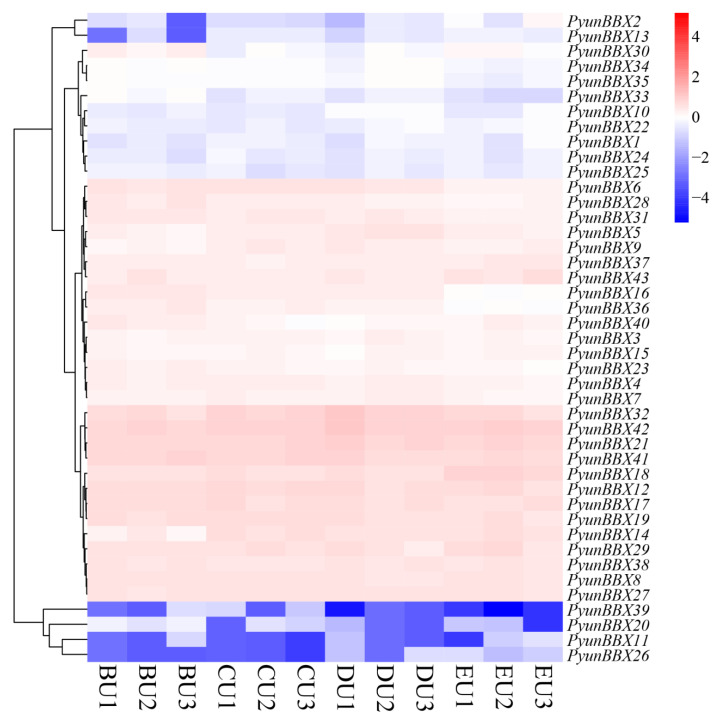
Gene expression levels of 43 *PyunBBX* genes in different parts of bark. The FPKMs of 43 *PyunBBX* genes are taken as the logarithm with base 10 for standardization; the color patches of different colors indicate the expression levels of *PyunBBX* genes in different parts of bark of *P. yunnanensis*. BU/CU/DU/EU: four positions of bark tissue: BU/CU: above and below the shoot site, DU: at the bottom of the new growth, EU: at the top (Figure S2). The number 1/2/3 represents three biological duplications. The connecting lines on the left represent cluster analysis.

The expression patterns of *PyunBBX* genes in leaf and bark were analyzed using RNA-seq data of leaves and bark in NCBI. Compared to the bark, *PyunBBX12/15/18/29/36/37/38* expression quantity is relatively high in the leaves. The expression level of *PyunBBX13* was very low in bark, with almost no expression (average of FPKM= 0.014062), while it was expressed in leaves (average of FPKM = 4.59). Compared with the leaves, *PyunBBX3/4/6/8/14/16/27/28/31/32/40* expressed in the bark of the organization’s quantity is relatively high (Figure 7). The expression patterns of *PyunBBXs* in bark tissues at different locations showed that the expression patterns of most *PyunBBXs* were basically consistent, indicating that they performed the same function in bark tissues at different locations (Figure S2) [52]. The expression level of *PyunBBX16/36/42* in the younger bark position (EU, Figure S2) was lower than that of other bark tissues, and the expression level of *PyunBBX18* in EU was higher than that of other bark tissues (Figure 8).

### 3.6. Expression Patterns of Six PyunBBXs under UV Treatment by RT-qPCR Analysis

**Figure 9:**
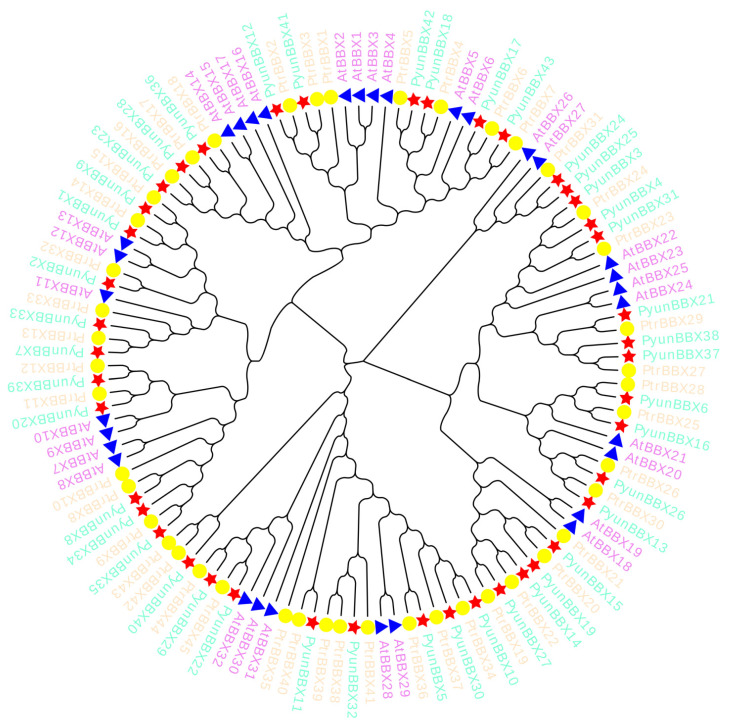
A rooted phylogenetic tree representing the relationships in *P. yunnanensis, A. thaliana,* and *P. trichocarpa*. We used the neighbor-joining (NJ) method with 1000 bootstrap replications to construct a phylogenetic tree. The *BBX* genes of a species are shown by genes name in the same colors and the same shapes in same colors (genes name in green and stars in red: *PyunBBX* genes of *P. yunnanensis*; genes name in orange and circles in yellow: *PtrBBX* genes of *P. trichocarpa*; genes name in purple and triangles in blue: *AtBBX* genes of *A. thaliana*).

**Figure 10:**
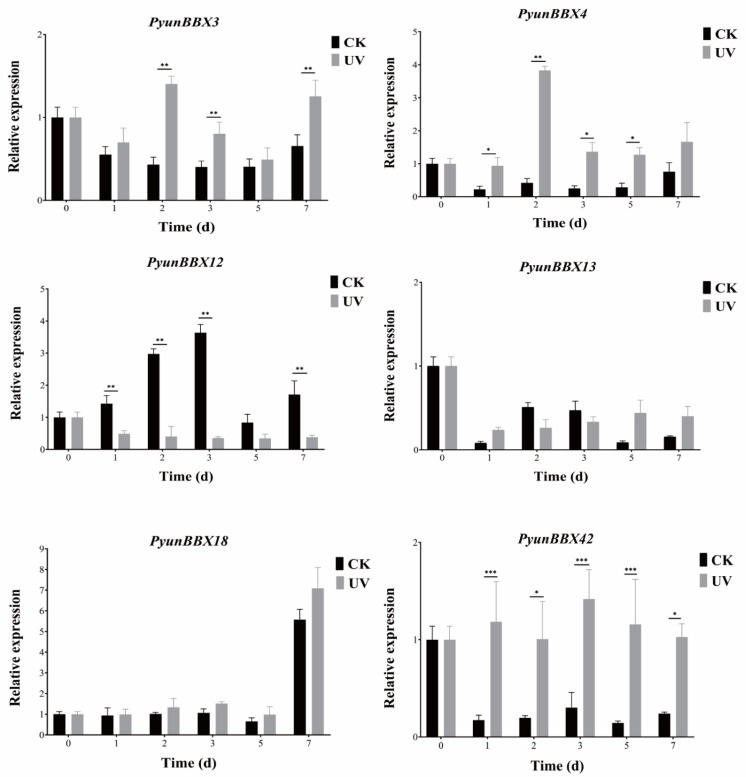
Relative expression analysis of six *PyunBBX* genes. CK: control check; UV: UV treatment; *: significant correlation at the 0.05 probability level; **: significant correlation at the 0.01 probability level; ***: significant correlation at the 0.001 probability level. Error bars indicate the SD of three independent biological and technical replicates. Time (d): days after UV treatment for 12 h a day.

**Figure 11:**
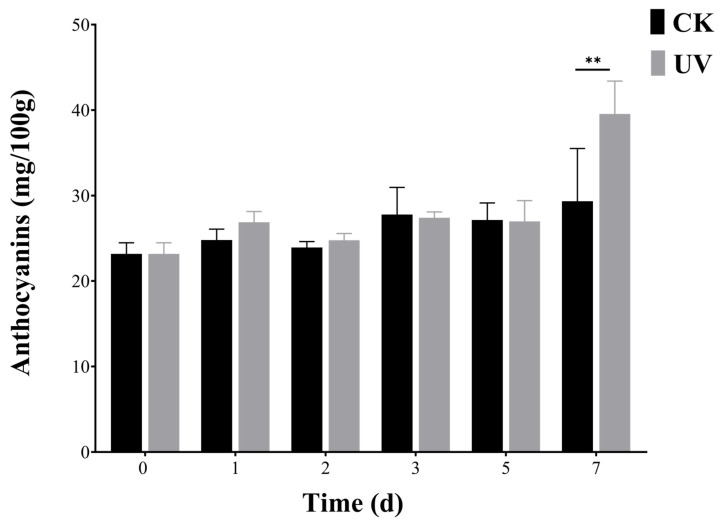
Anthocyanins content in leaves of *P. yunnanensis* in the six time periods. CK: control check; UV: UV treatment; **: significant correlation at the 0.01 probability level; Error bars indicate the SD of three independent biological and technical replicates. Time (d): days after UV treatment for 12 h a day.

In order to determine the *PyunBBX* genes in response to UV, we picked out the highly expressed *PtrBBXs* of *P. trichocarpa* under the UV-B treatment (*PtrBBX1/2/5/8/23/24/25/29*). Based on the relationship of homology and collinearity (Figure 9), *PyunBBXs* with high homology to these eight genes were selected from the *BBX* gene family of *P. yunnanensis* [12]. A total of 12 *PyunBBX* genes were obtained. In order to determine whether these 12 *PyunBBX* genes interact with MYB, *PyunBBX* genes with MYB binding sites were screened according to PlantCARE results, and finally, six *PyunBX* genes were determined (Figure S3, Table S2). Under UV treatment, the results of RT-qPCR showed that the six *PyunBBX* genes showed different expression patterns (Figure 10), and the relative expression level of *PyunBBX3/4/42* increased compared with the control group. The expression of *PyunBBX12* was inhibited, suggesting that *PyunBBX12* may be involved in the negative feedback regulatory pathway. The expression level of *PyunBBX18* was up-regulated on day 7, which was consistent with anthocyanins accumulation observed on day 7 (Figure 11), indicating that *PyunBBX18* may be directly involved in regulating the anthocyanins biosynthesis pathway. Compared with the control group, the expression level of *PyunBBX13* showed a high–low–high trend under UV treatment, indicating the complexity of *PyunBBX13* response to UV.

## 4. Discussion

### 4.1. Identification and Analysis of PyunBBX Gene Family Members in P. yunnanensis

In this study, 43 *BBX* gene family members were identified from the genome of *P. yunnanensis*, which can be divided into five types (Figure 3). They all have typical B-box structures, but the B-box1 structure of *PyunBBX5* lacks the amino acid sequence of C-X_2_-C (Figure S1). Therefore, *PyunBBX5* is a type of *BBX*-like gene, and its functions need to be further verified.

The process of segmental and tandem duplication is crucial for the amplification of gene family members in the genome during evolution [53]. In this study, we analyzed tandem and segmental duplication events to study the evolutionary path of *BBX* genes in *P. yunnanensis*. We found only segmental duplication in *BBX* genes (Table 2), suggesting that fragment duplication is involved in the expansion of *PyunBBX* genes.

In the phylogenetic tree (Figure 7) of *P. yunnanensis*, *P. trichocarpa,* and *A. thaliana*, it was found that *BBX* family genes were not clustered strictly according to the combination of conserved domains, and the uneven distribution may be due to a large number of genes or slight differences in domain organization in plant species.

The promoter regions of 43 *PyunBBX* genes also contain cis-acting elements of various plant hormones, such as ABA (abscisic acid), JA (jasmonic acid), IAA (indoleacetic acid), GA (gibberellic acid), ethylene, etc. Their functions in *P. yunnanensis* deserve further study.

In order to identify the UV-responsive *PyunBBX* genes under UV stress, we selected the up-regulated *BBX* genes of P. trichocarpa under UV treatment [12]. According to homology, we obtained 12 candidate *PyunBBX* genes. Finally, six *PyunBBX* genes were identified by the cis-acting elements of the promoter. The results of RT-qPCR showed that only the expression of *PyunBBX3/4/18/42* was increased under UV treatment, while the expression of *PyunBBX12* was inhibited. Moreover, *PyunBBX13* showed functional complexity. The above shows that homologous genes of near-origin species may have similarities and differences in performing functions.

There is a VP motif binding to *COP1* on the C segment of *PyunBBX18*, indicating that it interacts with *COP1*. In addition, *PyunBBX12/42* also has a VP motif, revealing that they are important factors involved in light signal transduction and regulatory pathways.

### 4.2. BBX Is Involved in Anthocyanins Biosynthesis

Structural genes encoding key enzymes in anthocyanins biosynthesis are regulated by transcription factors. Widely studied TFs include the *MYB* gene family, *bHLH* gene family, WD40 gene protein, etc., which regulate anthocyanins biosynthesis by combining with functional elements in the promoter region of structural genes. For example, overexpression of *SIMYB75* in tomatoes can increase anthocyanin content in fruits [54]. *MYB* TFs Aft can negatively regulate anthocyanins biosynthesis by binding to the promoter of negative regulator *SlMYBATV* [55]. Two *bHLH* TFs, *JAF13* and *AN1,* in petunias can directly or indirectly bind with MYB transcription factor *AN2* to activate *DFR* expression and promote anthocyanins biosynthesis [56,57]. The expression level of the *StWD40* gene in potatoes was significantly up-regulated in red and purple tubers, suggesting that the up-regulated expression level of *WD40* could promote anthocyanins accumulation [58].

Studies have shown that UV-B light can induce anthocyanin accumulation in plants. In apples, both *MdHY5* and *MdBBX22* could independently increase anthocyanin accumulation in the callus of apples under UV-B induction, and further investigation revealed that *MdBBX22* and *MdHY5* had interaction to jointly promote anthocyanin accumulation. In addition, both *MdBBX24* and *MdBBX33* interact with *MdHY5* to regulate anthocyanins biosynthesis [13], suggesting that *BBX-HY5* interaction is a universal regulatory module [8,59]. Recent studies have revealed that the interaction between *HY5* and the *BBX* TF family affects anthocyanin accumulation, and B-box proteins respond to light induction and assist HY5 proteins in participating in transcriptional regulation of anthocyanin biosynthesis in plants [60]. Therefore, whether *PyunBBX18* interacts with *HY5* to affect anthocyanin accumulation of *P. yunnanensis* under UV-B stress requires further experimental verification.

Flavonoids are usually accumulated in leaves, roots, and bark [61,62]. By analyzing RNA-seq data of leaves and bark of *P. yunnanensis*, *PyunBBX18* was found to be highly expressed in leaves and young bark, suggesting that *PyunBBX18* may be involved in the synthesis of flavonoids. According to PlantCARE prediction results, the promoter of *PyunBBX18* has three MYB binding sites (Figure S3), so it may interact with MYB TFs to regulate the synthesis of flavonoids.

## 5. Conclusions

In this study, 43 *PyunBBX* genes were identified and analyzed at the genome-wide level. They were distributed unevenly on 15 chromosomes, and their evolutionary relationships, physical and chemical properties, gene structure, and conserved motifs were analyzed. Collinearity analysis revealed that there were 14 pairs of homologous genes in the *P. yunnanensis BBX* gene family, and these genes were purified and selected. In addition, RNA-seq showed that *PyunBBX18* was highly expressed in leaves and younger bark. The results showed that *PyunBBX18* may be a key gene in UV-induced anthocyanin biosynthesis through the determination of anthocyanins content and the expression of genes.

## Figures and Tables

**Table 1:** Basic characteristic of *BBX* family members in *P. yunnanensis*.

Gene ID	Chromosomal Location	Amino Acid Length (aa)	Isoelectric Point (pI)	Molecular Weight (kDa)	Subcellular Localization
*PyunBBX1*	Chr01: 5,540,646–5,543,961	513	5.75	56.51	Nucleus
*PyunBBX2*	Chr01: 36,111,780–36,114,290	4 30	5.54	48.42	Nucleus
*PyunBBX3*	Chr01: 43,995,595–43,999,866	297	5.68	31.88	Nucleus
*PyunBBX4*	Chr01: 44,107,170–44,111,390	297	5.99	31.88	Nucleus
*PyunBBX5*	Chr01: 47,801,948–47,803,497	271	4.31	29.56	Nucleus
*PyunBBX6*	Chr02: 1,909,069–1,910,516	311	6.05	34.36	Nucleus
*PyunBBX7*	Chr02: 19,567,193–19571319	362	5.7	40.72	Nucleus
*PyunBBX8*	Chr02: 20,923,421–20,934,900	416	5.68	45.58	Nucleus
*PyunBBX9*	Chr03: 20,261,596–20,265,145	541	6.19	59.46	Nucleus
*PyunBBX10*	Chr04: 1,800,513–1,801,098	184	4.15	20.32	Nucleus
*PyunBBX11*	Chr04: 1,805,089–1,811,255	236	4.67	25.65	Nucleus
*PyunBBX12*	Chr04: 11,046,033–11,048,035	369	5.92	40.86	Nucleus
*PyunBBX13*	Chr04: 20,203,185–20,203,893	206	5.72	22.9	Nucleus
*PyunBBX14*	Chr04: 20,382,296–20,384,272	203	5.98	22.75	Nucleus
*PyunBBX15*	Chr05: 9,739,681–9,741,476	193	7.56	21.42	Nucleus
*PyunBBX16*	Chr05: 25,453,505–25,455,163	311	6.2	34.34	Nucleus
*PyunBBX17*	Chr06: 18,887,979–18,889,903	384	6.23	42.36	Nucleus
*PyunBBX18*	Chr06: 28,508,368–28,511,098	346	6.09	37.58	Nucleus
*PyunBBX19*	Chr07: 1,484,594–1,486,875	192	6.49	21.18	Nucleus
*PyunBBX20*	Chr07: 16,742,283–16,744,039	433	5.26	47.03	Nucleus
*PyunBBX21*	Chr07: 17,319,158–17,322,042	235	4.8	26.05	Nucleus
*PyunBBX22*	Chr08: 292,896–293,927	250	8.66	27.54	Nucleus
*PyunBBX23*	Chr08: 8,052,050–8,054,730	444	5.58	49.27	Nucleus
*PyunBBX24*	Chr08: 8,604,637–8,611,266	500	5.81	56.13	Nucleus
*PyunBBX25*	Chr08: 8,669,884–8,676,275	500	5.78	56.09	Nucleus
*PyunBBX26*	Chr09: 12,288,823–12,289,565	217	6.24	24.06	Nucleus
*PyunBBX27*	Chr09: 12,435,516–12,440,014	238	7.05	26.38	Nucleus
*PyunBBX28*	Chr10: 156,680,74–15,670,342	447	5.1	48.93	Nucleus
*PyunBBX29*	Chr10: 24,419,560–24,421,322	263	9.26	28.89	Nucleus
*PyunBBX30*	Chr11: 4,714,212–4,714,775	187	4.12	20.59	Nucleus
*PyunBBX31*	Chr11: 15,010,674–15,015,143	298	5.85	32.2	Nucleus
*PyunBBX32*	Chr11: 17,403,770–17,405,035	336	4.34	37.13	Nucleus
*PyunBBX33*	Chr14: 9,996,595–10,004,305	315	5.3	35.29	Nucleus
*PyunBBX34*	Chr14: 13,912,158–13,917,939	416	5.09	45.2	Nucleus
*PyunBBX35*	Chr14: 14,454,914–14,460,689	416	5.04	45.34	Nucleus
*PyunBBX36*	Chr15: 8,537,097–8,539,444	426	5.12	48.43	Nucleus
*PyunBBX37*	Chr17: 2,781,131–2,783,909	238	4.77	26.02	Nucleus
*PyunBBX38*	Chr17: 3,054,640–3,057,429	238	4.77	25.99	Nucleus
*PyunBBX39*	Chr17: 4,089,911–4,091,481	421	5.85	45.62	Nucleus
*PyunBBX40*	Chr17: 4,094,941–4,096,953	108	8.61	12.11	Nucleus
*PyunBBX41*	Chr17: 14,748,917–14,751,095	380	5.25	41.61	Nucleus
*PyunBBX42*	Chr18: 966,086–968,260	350	5.6	37.89	Nucleus
*PyunBBX43*	Chr18: 12,620,866–12,622,557	382	5.96	41.94	Nucleus

**Table 2:** Analysis of evolutionary selection pressure on co-linear members of *PyunBBX* genes.

Syntenic Gene Pairs	Method	Ks	Ka	Ka/Ks	Duplicated Type	Divergence Time (Mya.)
*PyunBBX22-PyunBBX29*	MA *	0.117574	0.350705	0.335249	Segmental	3.92
*PyunBBX15-PyunBBX19*	MA	0.0944959	0.335761	0.281438	Segmental	3.15
*PyunBBX13-PyunBBX26*	MA	0.0922884	0.265914	0.347061	Segmental	3.08
*PyunBBX1-PyunBBX9*	MA	0.0828391	0.301719	0.274557	Segmental	2.76
*PyunBBX23-PyunBBX28*	MA	0.0735978	0.217724	0.338032	Segmental	2.45
*PyunBBX4-PyunBBX31*	MA	0.0733725	0.226066	0.324562	Segmental	2.45
*PyunBBX14-PyunBBX27*	MA	0.0726848	0.378882	0.19184	Segmental	2.42
*PyunBBX20-PyunBBX39*	MA	0.0677018	0.192095	0.35244	Segmental	2.26
*PyunBBX17-PyunBBX43*	MA	0.0672206	0.355935	0.188856	Segmental	2.24
*PyunBBX12-PyunBBX41*	MA	0.0653516	0.3069	0.212941	Segmental	2.18
*PyunBBX6-PyunBBX16*	MA	0.0542511	0.285174	0.190239	Segmental	1.81
*PyunBBX21-PyunBBX37*	MA	0.0530882	0.237577	0.223457	Segmental	1.77
*PyunBBX18-PyunBBX42*	MA	0.0426761	0.358764	0.118953	Segmental	1.42
*PyunBBX34-PyunBBX35*	MA	0.00717976	0.0300601	0.238847	Segmental	0.24

* Model averaging.

## Data Availability

Genomic data of *P. yunnanensis* can be obtained by contacting the corresponding author. The data that support the findings of this study are available from the corresponding author upon reasonable request.

## Supplementary Materials

The following supporting information can be downloaded at: https://www.mdpi.com/article/10.3390/genes13101811/s1, Figure S1: Amino acid sequences of conserved domains (B-box1/B-box2, CCT, and VP) of *BBX* genes family in *P. yunnanensis*; Figure S2: Sampling positions on the cuttings of *P. yunnanensis*. Figure S3: Cis-acting elements of six *PyunBBX* genes; Table S1: Primers for RT-qPCR; Table S2: The results of PlantCARE for *PyunBBX* genes.
