# Supplementary material for: PyunBBX18 Is Involved in the Regulation of Anthocyanins Biosynthesis under UV-B Stress
Source: Genes (Basel). 2022 Oct 7;13(10):1811. doi: 10.3390/genes13101811 (PMC9602082; doi:10.3390/genes13101811)
Supplement: Supplementary file 1 [file genes-13-01811-s001.zip › Supplementary Materials/Supplementary Materials.pdf]

Table S1. Primers for RT-qPCR

Gene name	Forward Primer(5' to 3')	Reverse Primer(5' to 3')
PyunBBX3	ACCCATGTGTCTGCTCATCA	GGCCCAAATTCTCCAACTCC
PyunBBX4	CCGAAGAAGAGGTAGACGATGA	TAGAGTTGCTGCCGCCATT
PyunBBX12	GTGAGGAGGAGGAGGATGAAG	CTGCCGTTGCTGTTGATGAT
PyunBBX13	AGGTGTAAGGCTCTCTGCTTCT	CCGTTGTTGCTGATGCTGTCTC
PyunBBX18	GGATTTGGATCGGTATGTGG	ACCAAAGCAGGAGCTTGAAA
PyunBBX42	GGTTCGGAAATGGATTCGTA	TTACTTTGCACAGGCACCAC
HIS	TTTAAGACTGATCTGCGTTTCC	GAACAGCCCAACAAGGTATG

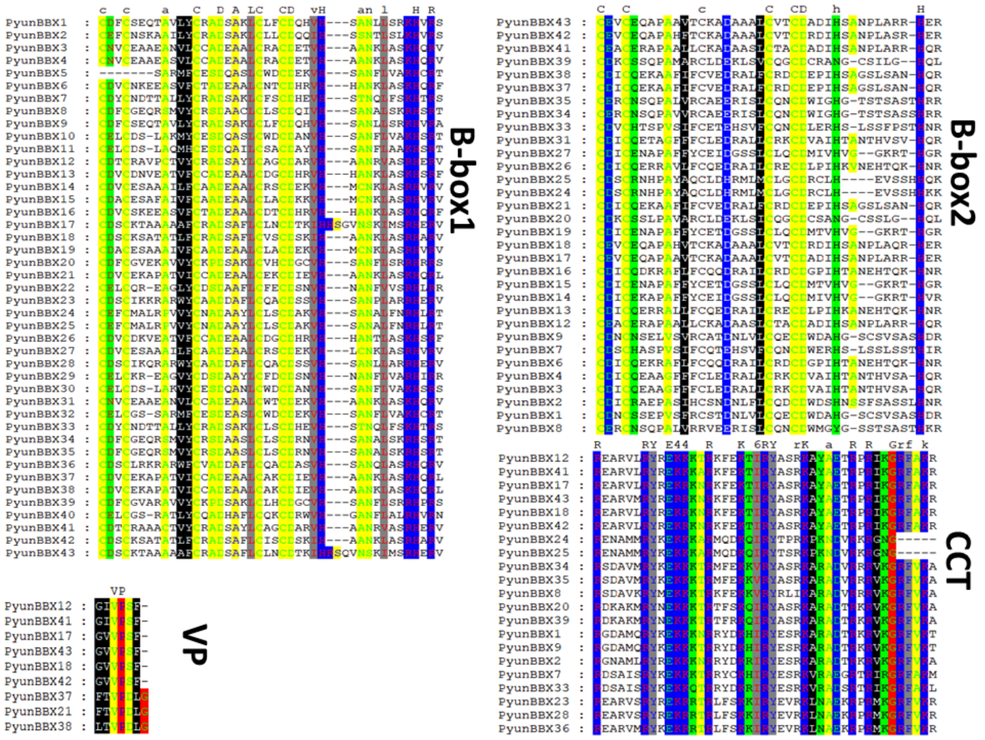

Figure S1. Amino acid sequences of conserved domains (B-box1/B-box2, CCT and VP) of *BBX* genes family in *P. yunnanensis*.

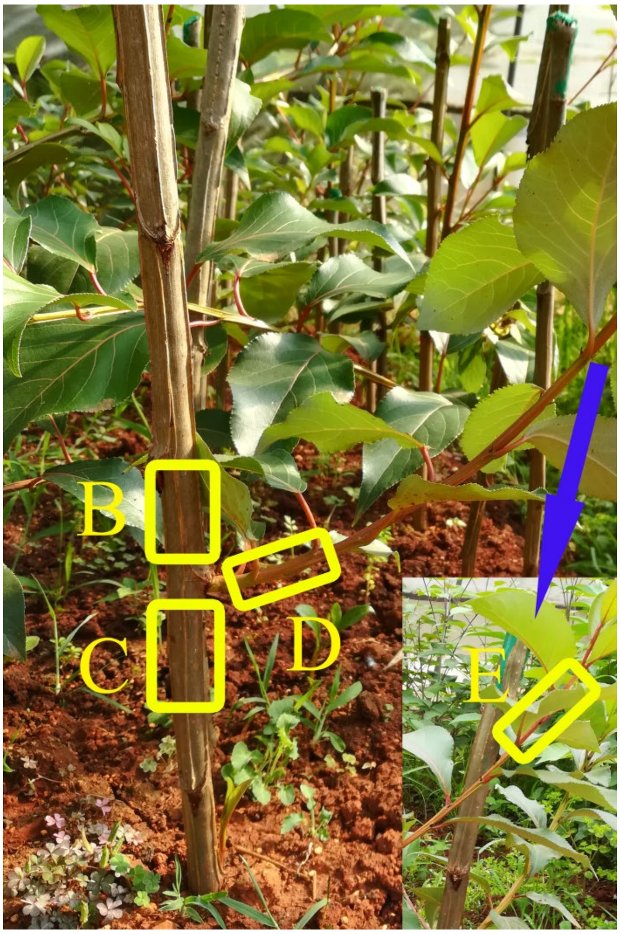

Figure S2. Sampling positions on the cuttings of *P. yunnanensis* [62] (Fig S1). B: BU, C: CU, D: DU, E: EU.

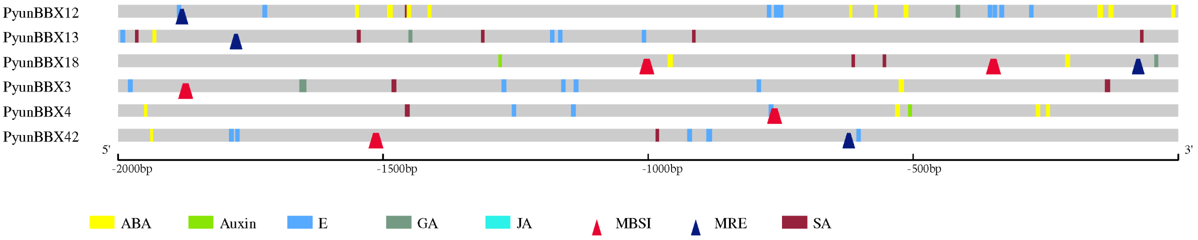

Figure S3. Cis-acting elements of six *PyunBBX* genes. ABA, Cis-acting elements of abscisic acid responsiveness, Auxin, Cis-acting elements of auxin responsiveness, E, Ethylene responsiveness, GA, Cis-acting elements of gibberelin responsiveness, JA, Cis-acting elements of jasmonic acid responsiveness, MBSI, MYB binding site involved in flavonoid biosynthetic genes regulation, MRE, MYB binding site involved in light responsiveness, SA, Cis-acting elements of salicylic acid responsiveness.
